# The protective effect of social support on all-cause and cardio-cerebrovascular mortality among middle-aged and older adults in the US

**DOI:** 10.1038/s41598-024-55012-w

**Published:** 2024-02-27

**Authors:** Yu Wang, Jun-Jun Wang, Hao-Feng Zhou, Wei-Ya Li, Ying-Xue Liao, Ming-Yu Xu, Chuan-Yu Gao, Bo Lv

**Affiliations:** 1 Fuwai Central China Cardiovascular Hospital, Zhengzhou, China; 2 Guangdong Cardiovascular Institute, Guangdong Provincial People’s Hospital, Guangdong Academy of Medical Sciences, Guangzhou, Guangdong China; 3 Institute of Cardiovascular Diseases, West China Hospital, Sichuan University, Chengdu, China; 4 Department of General Practice, Guangdong Provincial People’s Hospital (Guangdong Academy of Medical Sciences), Southern Medical University, Guangzhou, Guangdong China

**Keywords:** Social support, All-cause mortality, Cardio-cerebrovascular mortality, Middle-aged and older adults, NHANES, Psychology, Risk factors

## Abstract

The relationship between social support and mortality, especially cardio-cerebrovascular mortality, still has some limitations in the assessment of social support, sample selection bias, and short follow-up time. We used the data from 2005 to 2008 National Health and Nutrition Examination Survey to examine this relationship. The study analyzed a total of 6776 participants, divided into Group 1, Group 2, and Group 3 according to the social support score (0–1; 2–3; 4–5). Multivariable adjusted COX regression analyses of our study showed that Group 3 and Group 2 had a reduced risk of all-cause and cardio-cerebrovascular mortality (Group 3 vs 1, HR: 0.55, *P* < 0.001; HR: 0.4, *P* < 0.001; Group 2 vs 1, HR: 0.77, *P* = 0.017; HR: 0.58, *P* = 0.014) compared with Group 1. The same results were observed after excluding those who died in a relatively short time. Additionally, having more close friends, being married or living as married, and enough attending religious services were significantly related to a lower risk of mortality after adjustment. In brief, adequate social support is beneficial in reducing the risk of all-cause mortality and cardio-cerebrovascular mortality in middle-aged and older adults, especially in terms of attending religious services frequency, the number of close friends, and marital status.

## Introduction

Social support generally refers to the emotional, instrumental and informational assistance that people can use or that is actually provided to them. It may be measured objectively by marital status, contacts with friends and relatives, religious and group membership, as well as subjectively through perceived help from others^1^. Social support can positively affect the cardiovascular, immune, metabolic, and nervous and endocrine systems by promoting healthy behaviors, such as good eating habits and physical exercise^2–5^. Previous studies have shown that middle-aged and older populations tend to have reduced social support, and lower social support is associated with poorer physical and mental health^6^. Numerous studies have indicated that the lack of social support is associated with an increased risk of incident coronary heart disease, depression, as well as cognitive and functional decline^7–10^.

The impact of social support on mortality is increasingly drawing attention. A large number of studies demonstrated that social support is independently associated with all-cause mortality in multiple populations after adjusting for demographic characteristics and initial health status^11–15^, although a few studies reported no significant relation between the two^16,17^. But studies performed to date have some methodological limitations, including the use of oversimplified questionnaires without evaluating reliability and validity^13,14,16^, and limited sample sizes or short follow-up time periods^11,12^. More robust evidence is needed to illustrate the relationship between social support and mortality.

National Health and Nutrition Examination Survey (NHANES), founded by National Institutes of Health, recruited participants all over the United States, and the questionnaire of social support adapted in NHANES was developed from large-scale community epidemiological studies, gathering information on social support from five aspects: emotion, finance, marital status, number of friends and religious membership^18–20^. Therefore, our aim is to utilize data from NHANES to examine whether social support independently predicts all-cause mortality in the middle-aged and older population. Additionally, we explore the relationship between social support and cardio-cerebrovascular mortality, considering that cardio-cerebrovascular disease remains the leading cause of morbidity and mortality globally. This study has the potential to advance public awareness on social health and promote additional protection of vulnerable population.

## Methods

### Study design and study population

Our study aimed to investigate the influence of social support on mortality in the general US adult population using NHANES data. NHANES is a cross-sectional continuous series research aims to monitor the health of the US population. A complex, multistage, probability sampling design was used to select individual representative of the US population.

**Figure 1:**
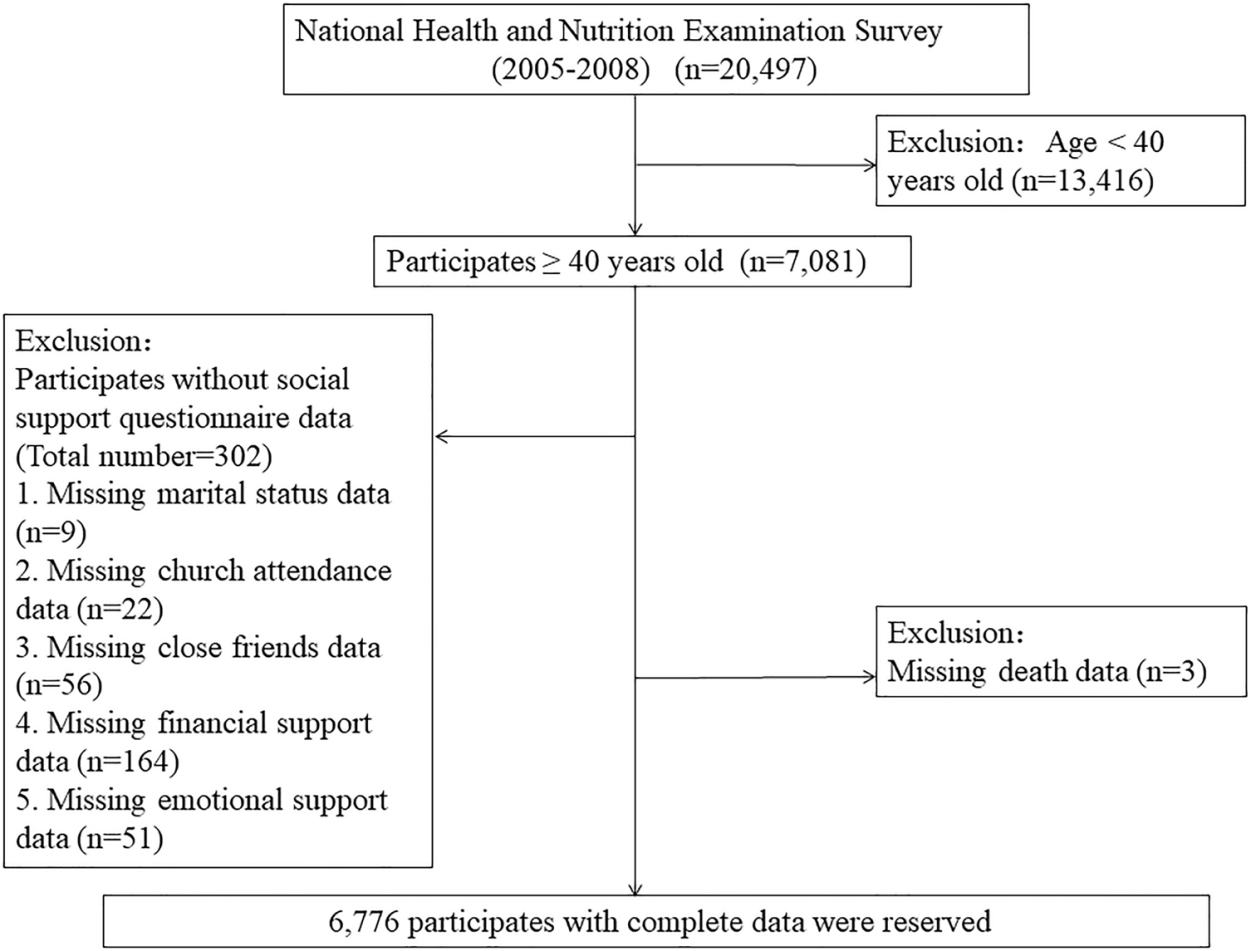
The research flow chart.

The population of interest included those participates who finished the social support questionnaire in the 2005–2008 NHANES. Only participates who were 40 years old and above were qualified to answer the NHANES social support questionnaire. Trained household interviewers administered all the questionnaires in the sample participant’s home. Participants under the age of 40 were initially excluded. Subsequently, participants for whom the total social support score could not be calculated due to any missing social support components were excluded. Individuals with the missing information of death was also excluded. Finally, a total of 6776 individuals entered in the analysis. The flow chart of sample screening was showed in the Fig. 1. The report was in accordance with the Strengthening the Reporting of Observational Studies in Epidemiology (STROBE) guidelines for cohort studies.

### Ethical approval and informed consent

The study protocols of NHANES were approved by the National Center for Health Statistics (NCHS) institutional review board and all the participants signed a written informed consent^21,22^. Ethical review and approval were waived for our study, since all the data from NHANES was publicly accessible. All the details of enrollment, procedures, and other data for NHANES can be acquired by visiting https://www.cdc.gov/nchs/nhanes/index.htm.

### Social support assessment

We use five dichotomous social support variables to form a social support scoring index. The structure and predictive effectiveness of this index for NHANES have been proven in many previous studies^3–5^. The five aspects of social support scoring index respectively are emotional support, financial support, frequency of attending religious services, number of close friends, and marital status. Specifically, 1 point was assigned to the answer “yes” for the question on the emotional support (Can you count on anyone to provide with emotional support such as talking over problems or helping make a difficult decision?) and on the financial support (Could you count on anyone to help, for example, by paying any bills, housing costs, hospital visits, or providing with food or clothes?). The answers “no”, “don’t need” or “don’t accept” were assigned as “0”. 1 point was assigned for married or living as married, attending at least four religious services per year (How often do you attend church or religious services?), and having four or more close friends (In general, how many close friends do you have?). In any of these questions, participates who refused to answer the question or answered “don’t know” were considered as missing data. Because these data could not be defined as either having or missing social support. The sum of social support score ranged from 0 to 5.

### Covariates

Age was divided into five categories of 40–49, 50–59, 60–69, 70–79, and 80 years and older. Body mass index (BMI) was calculated as weight divided by height (kg/m^2^). Education was recorded as four levels including less than high school graduate, high school graduate or equivalent, some college or associate of arts degree, and college graduate or above. Race was described as non-Hispanic White; Mexican American and other Hispanic; non-Hispanic Black; Other. Using the American Heart Association (AHA) secondary diet score to estimate dietary management; eight dietary elements were included, with a total score of 80. A score < 32 was considered to indicate a poor diet^23^. We combined three questions from the NHANES to determine whether participants smoked or not. Individuals who answered “yes” to any question of “Have you smoked at least 100 cigarettes in life?”, “Do you now smoke cigarettes?” and “Used tobacco/nicotine last 5 days?” were divided into smoking group. Hypertension was defined as the patient admitting to having a history of high blood pressure.

### Mortality data

The primary outcomes of interest included all-cause and cardio-cerebrovascular death until December 31, 2015. Mortality data were extracted from the mortality file which involved a probabilistic match between NHANES and National Death Index records provided by the NCHS. International Classification of Diseases, Tenth Revision codes (I00-I09, I11, I13, I20-I51, I60-I69) were used to define cardio-cerebrovascular deaths. All-cause mortality was defined as death from any cause. The median follow-up time in our study was 96.5 ± 27.7 (mean ± standard deviation) months.

### Statistical analyses

Statistical analysis for this study was completed using SPSS version 25 and all the level of significance was set at *P* < 0.05. The participates were divided into 3 groups according to the social support score level (Group 1: social support score = 0–1; Group 2: social support score = 2–3; Group 3: social support score = 4–5). Our study mainly contained three parts: (1) Comparison of baseline characteristics in the three social support groups; (2) Next, we assessed the predict effect of social support on all-cause and cardio-cerebrovascular mortality in different models and subgroups; (3) Finally, the association between all the individual social support variables and all-cause as well as cardio-cerebrovascular mortality after multi-variable adjustment.

Continuous and classified variables were described as means (standard error), and number (percentage) respectively. The continuous variables including BMI and AHA secondary diet score are normally distributed, and their probability-probability (P-P) plots are shown in the Supplementary Fig. 1 and 2. Therefore, one-way analysis of variance (ANOVA) was utilized to analyze these differences among different groups. When analyzing categorical variables, the Chi-squared test was employed for different social support groups.

Variables with an obvious difference at baseline (*P* < 0.05) in the different social support groups were executed multivariate Cox regression analyses. Variable (gender and cancer or malignancy history) which was considered clinically associated with the mortality was also included in the multivariate Cox regression analysis. Finally, full adjusted variables included age, sex, BMI, education level, race, smoking, AHA Secondary Diet Score, cancer or malignancy history, diabetes, cardiovascular history, and cerebrovascular history. Multivariate Cox regression models were used to calculate the hazard ratios (HR) to estimate the association between social support levels and mortality after confounding variables adjustment. We also assessed the association between the five single social support variable and mortality by the Cox regression models. In order to avoid the bias of the early mortality caused by new major disease or accidents on our study, we extra executed sensitivity analyses after excluding participants who died within 1 year after the interview.

### Ethical standards

All procedures performed in studies involving human participants were in accordance with the ethical standards of the institutional and/or national research committee and with the 1964 Helsinki declaration and its later amendments or comparable ethical standards. This article does not contain any studies with animals performed by any of the authors. Written informed consent was obtained from all men and women who participated in the NHANES study.

## Results

### Baseline characteristics

Table 1 showed the baseline characteristics of this study population. A total of 6776 participants in NHANES from 2005 to 2008 were included in our final analyses. Of these 6776 people, 3451 (50.9%) were female, 3325 (49.1%) were male. 3508 (51.8%) were non-Hispanic white, 1476 (21.8%) were non-Hispanic black, 1511 (22.9%) were Mexican American and other Hispanic. 856 (12.6) had cardiovascular disease history, and 413 (6.1%) had cerebrovascular disease history. The majority of the sample were aged between 40 and 69 years (71.8%), less than high school graduate 2149 (31.7%). 3567 participants were divided into smoking group. The average of AHA Secondary Diet Score was 38.3 points and 1963 (29.0%) had a poor diet. 878 (13.0%) and 1122 (16.9%) had cancer or malignancy history and diabetes.

**Table 1:** Descriptive statistics by social support score of general adults in NHANES.

Variables	Total (n = 6776)	Social support score			*P*
		Group 1: 0–1 (n = 400)	Group 2: 2–3 (n = 2735)	Group 3: 4–5 (n = 3641)	
Age, range, n (%)£					**< 0.001**
40–49	1751 (25.8)	99 (24.8)	700 (25.6)	952 (26.1)	
50–59	1518 (22.4)	88 (22.0)	660 (24.1)	770 (21.1)	
60–69	1598 (23.6)	110 (27.5)	626 (22.9)	862 (23.7)	
70–79	1150 (17.0)	56 (14.0)	419 (15.3)	675 (18.5)	
≥ 80	759 (11.2)	47 (11.8)	330 (12.1)	382 (10.5)	
Male, n (%)£	3325 (49.1)	203 (50.7)	1352 (49.4)	1770 (48.6)	0.64
BMI (kg/m^2^), mean (SD)^a^	29.2 ± 6.5	28.7 ± 6.0	29.4 ± 7.2	29.1 ± 6.0	**0.035**
Education level, n (%)£					**< 0.001**
Less than high school graduate	2149 (31.7)	197 (49.3)	1062 (38.8)	890 (24.4)	
High school graduate/GED or equivalent	1646 (24.3)	97 (24.3)	667 (24.4)	882 (24.2)	
Some college or AA degree	1663 (24.5)	68 (17.0)	641 (23.4)	954 (26.2)	
College graduate or above	1312 (19.4)	38 (9.5)	361 (13.2)	913 (25.1)	
Race, n(%)£					**< 0.001**
Mexican American and other Hispanic	1511 (22.9)	132 (33.0)	707 (25.9)	712 (19.6)	
Non-Hispanic white	3508 (51.8)	184 (46.0)	1257 (46.0)	2067 (56.8)	
Non-Hispanic black	1476 (21.8)	73 (18.3)	653 (23.9)	750 (20.6)	
Other race	241 (3.6)	11 (2.8)	118 (4.3)	112 (3.1)	
Smoking, n (%)£	3567 (52.6)	262 (65.5)	1561 (57.1)	1744 (47.9)	**< 0.001**
AHA secondary diet score, mean (SD)^a^	38.3 ± 12.1	36.5 ± 12.2	37.6 ± 12.0	39.1 ± 12.1	**< 0.001**
Poor diet, n (%)£	1963 (29.0)	137 (34.3)	841 (30.7)	985 (27.1)	**< 0.001**
Hypertension, n (%)£	1516 (22.4)	83 (20.8)	605 (22.1)	828 (22.7)	0.61
Cancer or malignancy, n (%)£	878 (13.0)	49 (12.3)	346 (12.7)	483 (13.3)	0.71
Diabetes, n (%)£	1122 (16.9)	72 (18.5)	508 (19.0)	542 (15.2)	**< 0.001**
Cardiovascular disease history, n (%)£	856 (12.6)	60 (15.0)	374 (13.7)	422 (11.6)	**0.015**
Cerebrovascular disease history, n (%)£	413 (6.1)	32 (8.0)	190 (6.9)	191 (5.2)	**0.004**

*NHANES* National Health and Nutrition Examination Survey, *GED* General Education Development, *AA* associate of arts, *BMI* body mass index, *AHA* American Heart Association, *SD* standard deviation.

^a^For one-way analysis of variance test, £ for Chi-squared test. Bold characters mean a statistical significance.

A total of 6183 (91.2%) individuals had emotional support, and 4964 (31.7%) owed the financial support. 3972 (58.6%) attended religious services 4 times or more. 4262 (62.9%) had 4 or more close friends in our sample. And 4179 (61.7%) participates got 1 score for the marriage status (Supplementary Table 1). Finally, according to the total social support score, 400 (5.9%), 2735 (40.4%), 3641 (53.7%) were divided into Group 1 (Low social support), Group 2 (Medium social support), and Group 3 (High social support), respectively.

### Main results

**Figure 2:**
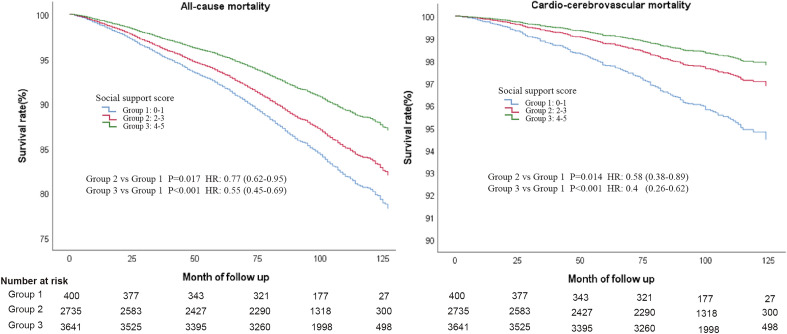
Cox regression curves for all-cause and cardio-cerebrovascular mortality according social support groups after full variable adjustment.

After a median follow-up of about 8 years, 1347 (19.9%) and 265 (3.9%) experienced all-cause and cardio-cerebrovascular mortality, respectively. In the Cox regression analysis of different models, individuals with high social support score (Group 3) always had the lower risk of all-cause and cardio-cerebrovascular mortality than those with low social support in all the different Models (Table 2, Fig. 2).

**Table 2:** Cox multivariate proportional hazard ratios for all-cause and cardio-cerebrovascular mortality by social support score in different models.

Variable	All-cause mortality		Cardio-cerebrovascular mortality	
	HR	95%CI	HR	95%CI
Model 1: Only social support score by itself				
Group 1(Social support score = 0–1)	Ref		Ref	
Group 2 (Social support score = 2–3)	0.82	0.67–1.01	0.69	0.45–1.05
Group 3 (Social support score = 4–5)	**0.56**	**0.46–0.7**	**0.43**	**0.28–0.66**
Model 2: Additionally adjusted for demographics^a^				
Group 1 (Social support score = 0–1)	Ref		Ref	
Group 2 (Social support score = 2–3)	0.83	0.67–1.02	0.69	0.45–1.06
Group 3 (Social support score = 4–5)	**0.56**	**0.45–0.69**	**0.43**	**0.28–0.66**
Model 3: Additionally adjusted for demographics and other covariates^b^ (Full variable adjustment)				
Group 1 (Social support score = 0–1)	Ref		Ref	
Group 2 (Social support score = 2–3)	**0.77**	**0.62–0.95**	**0.58**	**0.38–0.89**
Group 3 (Social support score = 4–5)	**0.55**	**0.45–0.69**	**0.40**	**0.26–0.62**
Model 3: Subgroup analyses: limit sample to:				
*Age*	P for interaction for all-cause and cardio-cerebrovascular mortality = 0.11 and 0.67			
Participants < 65 years				
Group 1 (Social support score = 0–1)	Ref		Ref	
Group 2 (Social support score = 2–3)	0.78	0.55–1.12	**0.46**	**0.23–0.94**
Group 3 (Social support score = 4–5)	**0.45**	**0.31–0.66**	**0.34**	**0.16–0.73**
Participants ≥ 65 years				
Group 1(Social support score = 0–1)	Ref		Ref	
Group 2 (Social support score = 2–3)	0.90	0.69–1.18	0.75	0.43–1.3
Group 3 (Social support score = 4–5)	**0.67**	**0.51–0.88**	**0.49**	**0.28–0.86**
*Gender*	P for interaction for all-cause and cardio-cerebrovascular mortality = 0.21 and 0.19			
Male				
Group 1 (Social support score = 0–1)	Ref		Ref	
Group 2 (Social support score = 2–3)	**0.71**	**0.54–0.94**	**0.58**	**0.34–0.99**
Group 3 (Social support score = 4–5)	**0.49**	**0.37–0.64**	**0.33**	**0.19–0.58**
Female				
Group 1 (Social support score = 0–1)	Ref		Ref	
Group 2 (Social support score = 2–3)	0.91	0.64–1.30	0.60	0.29–1.23
Group 3 (Social support score = 4–5)	**0.70**	**0.49–0.99**	0.50	0.24–1.04
Participants who died > 1 year after the interview				
Group 1 (Social support score = 0–1)	Ref		Ref	
Group 2 (Social support score = 2–3)	**0.75**	**0.60–0.94**	**0.59**	**0.37–0.95**
Group 3 (Social support score = 4–5)	**0.54**	**0.43–0.68**	**0.42**	**0.26–0.68**

Bold characters mean a statistical significance.

*CI* confidence interval, *HR* hazard ratios, *Ref* Reference.

^a^Demographic variables for mortality included age, sex, body mass index (BMI), education level, and race.

^b^Other covariates included smoking, diet condition, diabetes, cancer or malignancy history, cardiovascular disease history and cerebrovascular disease history.

**Figure 3:**
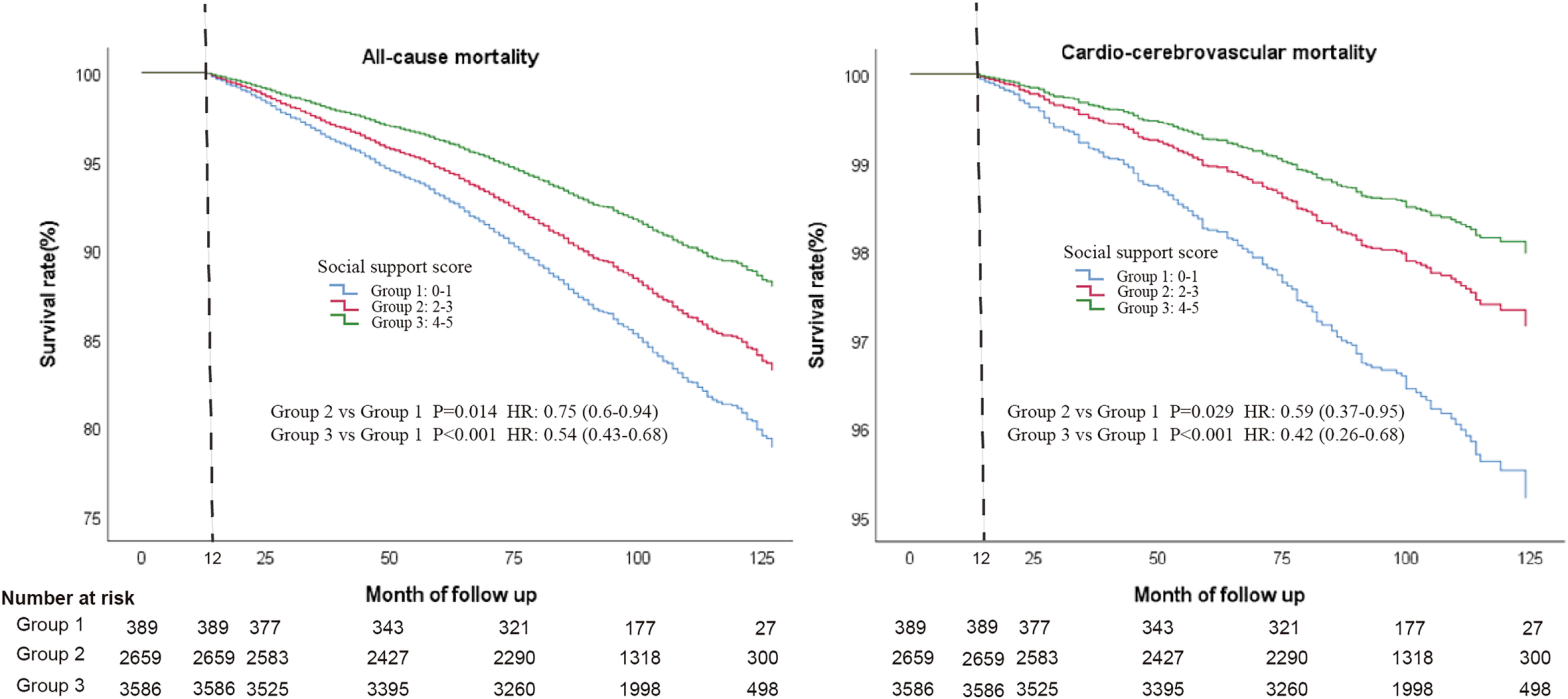
Cox regression curves for all-cause and cardio-cerebrovascular mortality according social support groups after full variable adjustment and excluding participants who died within a year of the interview.

After full variable adjustment (Including: age, sex, BMI, education level, race, smoking, AHA Secondary Diet Score, cancer or malignancy history, diabetes, cardiovascular history, and cerebrovascular history), Group 3 with a high social support could lower 45% all-cause mortality [Adjusted hazard ratios (aHR) = 0.55, 95% confidence interval (CI):0.45–0.69] and 60% cardio-cerebrovascular mortality (aHR = 0.40, 95% CI 0.26–0.62) than Group 1 with low social support, respectively. And Group 2 also had a significantly lower risk of all-cause (aHR = 0.77, 95% CI 0.62–0.95) and cardio-cerebrovascular mortality than Group 1 (aHR = 0.58, 95% CI 0.38–0.89). In the subgroup analysis, no significant difference was found in the age and gender subgroups (All the *P* for interactions > 0.05). After excluding those who died within a year after the interview, regardless of all-cause or cardio-cerebrovascular mortality, both Group 2 and Group 3 had significantly higher survival rate than Group 1 (For all-cause mortality: Group 2 vs Group 1, aHR = 0.75, 95% CI 0.60–0.94; Group 3 vs Group 1, aHR = 0.54, 95% CI 0.43–0.68; For cardio-cerebrovascular mortality: Group 2 vs Group 1, aHR = 0.59, 95% CI 0.37–0.95; Group 3 vs Group 1, aHR = 0.42, 95% CI 0.26–0.68; Fig. 3). All in all, the improvement of social support for reducing the cardio-cerebrovascular mortality is better than that of all-cause mortality.

Finally, we assessed the association between the individual social support variables and mortality. The five components were all considered dichotomous variables. In the Cox regression analysis, more close friends (For all-cause mortality: aHR = 0.87, 95% CI 0.77–0.98; For cardio-cerebrovascular mortality: aHR = 0.76, 95% CI 0.58–0.99) and married or living as married (For all-cause mortality: aHR = 0.75, 95% CI 0.67–0.85; For cardio-cerebrovascular mortality: aHR = 0.70, 95% CI 0.53–0.91) were significantly related to the lower risk of all-cause and cardio-cerebrovascular mortality after full variable adjustment. Attending more religious services was also associated with all-cause mortality (aHR = 0.74, 95% CI 0.66–0.83) of statistical significance. As for emotional and financial support, a decreased risk of all cause as well as cardio-cerebrovascular mortality was also observed, but didn’t reach statistically significant (Table 3).

**Table 3:** Cox proportional hazard ratios for all-cause and cardio-cerebrovascular mortality by individual social support variables after full variable adjustment.

Individual social support variable	All-cause mortality			Cardio-cerebrovascular mortality		
	HR	95%CI	*P* value	HR	95%CI	*P* value
Emotional support	0.97	0.84–1.26	0.77	0.88	0.58–1.34	0.56
Financial support	0.92	0.81–1.06	0.25	0.87	0.65–1.17	0.36
Attending religious services frequency ≥ 4/year	**0.74**	**0.66–0.83**	**< 0.001**	0.87	0.67–1.13	0.30
Number of close friends ≥ 4	**0.87**	**0.77–0.98**	**0.02**	**0.76**	**0.58–0.99**	**0.049**
Marital status	**0.75**	**0.67–0.85**	**< 0.001**	**0.7**	**0.53–0.91**	**0.008**

*CI* confidence interval, *HR* hazard ratios.

Full variable adjustment included age, sex, body mass index (BMI), education level, race, smoking, diet condition, diabetes, cancer or malignancy history, cardiovascular disease history and cerebrovascular disease history.

Bold characters mean a statistical significance.

## Discussion

Through the analysis of a large number of sample data from the nationally representative NHANES database, which has been followed up for a long time, it is found that all-cause mortality and cardio-cerebrovascular mortality are negatively related to the higher levels of social support. Adequate social support can lead to a reduction in the risk of all-cause mortality by up to 45% and cardio-cerebrovascular mortality by 60%. As the impact of social support on health is a long-term process, we conducted further analysis by excluding patients who died within 1 year of data collection. Since these patients may have died from emergencies or major new diseases, the results still suggest that the lower risk of all-cause mortality as well as cardio-cerebrovascular mortality are related to the adequate social support. Our research conclusions comply with previous research^24,25^, while more variables are included to have a more comprehensive reflection of the level of social support. Then, the relationship between each sub-variable that constitutes the total score of social support and the risk of death is further analyzed, thereby more comprehensively embodying the long-term potential effects of social support on death.

As in previous studies, the variables we selected included major aspects of perceived social support^19^. However, numerous confounding factors influence the impact of the social support level in predicting the mortality risk model, including demographic characteristics, lifestyle habits (such as smoking and diet), history of diabetes, tumor, cardiovascular and cerebrovascular diseases^19,24–26^. Adjustment of potential confounding factors is also an important work to improve the quality of the model. When constructing this prediction model, we first compared the baseline data of the variables reported in previous research that may affect the results, and further adjusted the variables with statistical differences. Therefore, the results were relatively accurate, indicating that this model holds significant value in predicting mortality risk and can serve as a reference for future research in the field of social support.

From our research results, it indicates that the participation in religious activities more than 4 times per year can reduce the risk of all-cause mortality by 28%. This data may differ from the results of previous studies, in which the different period of the study may be the reason^27,28^. However, the research conclusions remain consistent, affirming that active participation in religious activities contributes to the reduction of the risk of death. During the analysis, we checked potential confounding factors such as demographic data and the previous history of cardio-cerebrovascular diseases. Still, there may be mediators or confounding factors affecting the results, such as the timing of religious activities and individual self-discipline. In future research, it is essential to fully consider these aspects to further explore the causal relationship between participating in religious activities and physical health.

In accordance with our research results, it also indicates that the large number of friends and having partners (married or living with partner) also have negative correlation with all-cause mortality as well as cardio-cerebrovascular mortality, which comply with the conclusions of previous studies^29,30^. The social isolation of participants maybe increased by the lack of friends or partners. On the other hand, living alone may lead to a delay in obtaining acute care. Recently published meta-analyses have also confirmed that social isolation and loneliness can increase the risk of death^31,32^.

People who are socially isolated have more cumulative negative emotions or lack of emotional support, both of which are detrimental to cardiovascular health and increase the risk of death^33,34^. However, our statistical results do not reveal a connection between adequate emotional support and a lower risk of death. In fact, groups with sufficient emotional support exhibit a higher risk of all-cause mortality, although this difference is not statistically significant. The possible reason for this discrepancy is that the variable of emotional support relies on the subjective judgment of the subjects. Due to varying individual needs for emotional support, the results are likely affected. Previous studies also suggest that different sources of emotional support may lead to different results^29,33^. Due to the failure of the participants of this research to further clarify the source of emotional support, the conclusion may also be affected. In line with our research results, it suggests that adequate financial support does not have connection with the risk of all-cause mortality or cardio-cerebrovascular mortality, while it can’t be assumed that the two are unrelated. The adequate financial support can promote health by relieving mental stress and reduce the occurrence of adverse events such as death, which is supported by most previous studies^35^. However, the potential factors that affect the results can be reflected in individual's demand for economic support and economic differences in different regions. The above factors cannot be considered by the subjects in this research, which may be the reasons for the acquisition of different conclusions. In the future research, the full consideration should be given to draw more accurate conclusions.

Our statistical results indicate a negative correlation between the level of social support and all-cause mortality as well as cardio-cerebrovascular mortality. Based on this result, we divided the level of social support into three groups, which was also similar to the previous research methods^19^. Following the grouping, the results further demonstrated that a low social support level was associated with a high risk of all-cause mortality and cardio-cerebrovascular mortality. Moreover, with the increase in social support level, the hazard ratio (HR) value decreased simultaneously. Among these findings, the median level of social support revealed a gender difference in predicting the risk of all-cause mortality: the reduction in all-cause mortality risk was statistically significant only for men. Studies have indicated that women tend to provide more social support and are more susceptible to adverse events such as depression, myocardial ischemia, and even sudden death when lacking social support or experiencing shock^36,37^. These aspects reflect women's high need for social support, which could explain why the gender difference above disappeared in the group with high level of social support. These findings may also help understand how to improve quality of life from a social support perspective, but more research is needed to explore why social support affects mortality risk.

The advantage of this research lies in the large number of samples, with a nationally representative population and an extended follow-up time. The level of social support obtained by the subjects is comprehensively considered from multiple perspectives, and subgroup analysis for potential impactful factors is conducted (excluding individuals who died within 1 year). Moreover, efforts are made to correct potential confounding factors as much as possible, leading to reliable conclusions. However, limitations do exist. The data for this research spans from 2005 to 2008, introducing a potential discrepancy with the current situation. Additionally, data acquisition relies partly on questionnaires, which are highly subjective and may potentially influence the conclusions. Nevertheless, numerous previous studies have validated the validity and rationality of this questionnaire, ensuring the strong credibility of our research results. The assessment is solely made for the baseline social support situation, lacking dynamic tracking—a notable drawback of the NHANES database. Some variables that could introduce bias to the study results, such as alcohol intake, hyperlipidemia, cognitive impairment, and physical activity, couldn't be analyzed due to a large number of missing values, representing a shortcoming of this study. However, we adjusted all complete and essential covariates as thoroughly as possible to minimize bias in the study results. Lastly, the results obtained using the NHANES database may not be applicable to other countries or regions. The future development of a prospective study to continuously evaluate social support or explore the relationship between changes in social support and adverse events is deemed necessary.

## Conclusion

Adequate social support is beneficial for reducing the risk of all-cause mortality and cardio-cerebrovascular mortality, especially in terms of attending religious services frequency, the number of close friends, and marital status. Furthermore, conducting a prospective study to continuously evaluate the situation of social support or explore the relationship between changes in social support and adverse events is still necessary in the future.

### Supplementary Information

Supplementary Information.

## Data Availability

All the details of enrollment, procedures, and other data for NHANES can be acquired by visiting https://www.cdc.gov/nchs/nhanes/index.htm.
