# Supplementary material for: The protective effect of social support on all-cause and cardio-cerebrovascular mortality among middle-aged and older adults in the US
Source: Sci Rep. 2024 Feb 27;14:4758. doi: 10.1038/s41598-024-55012-w (PMC10899207; doi:10.1038/s41598-024-55012-w)
Supplement: Supplementary file 1 — Supplementary Information. [file 41598_2024_55012_MOESM1_ESM.pdf]

Supplementary Figure 1 Probability-probability plot of body mass index.

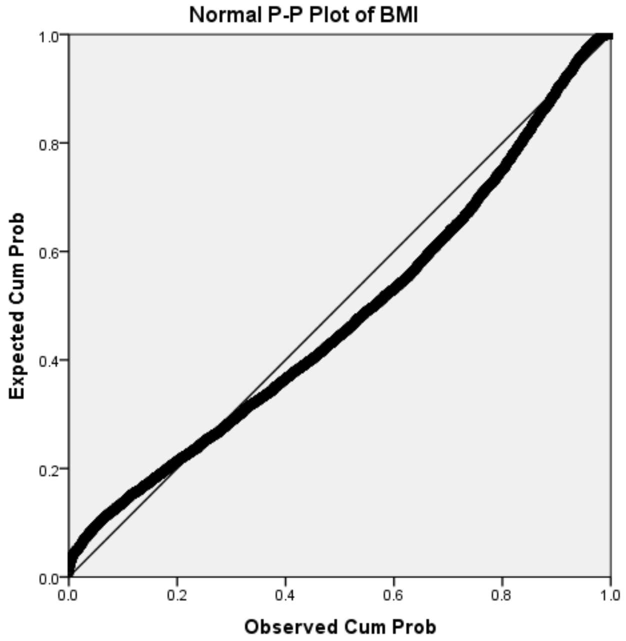

Supplementary Figure 2 Probability-probability plot of American Heart Association secondary diet score.

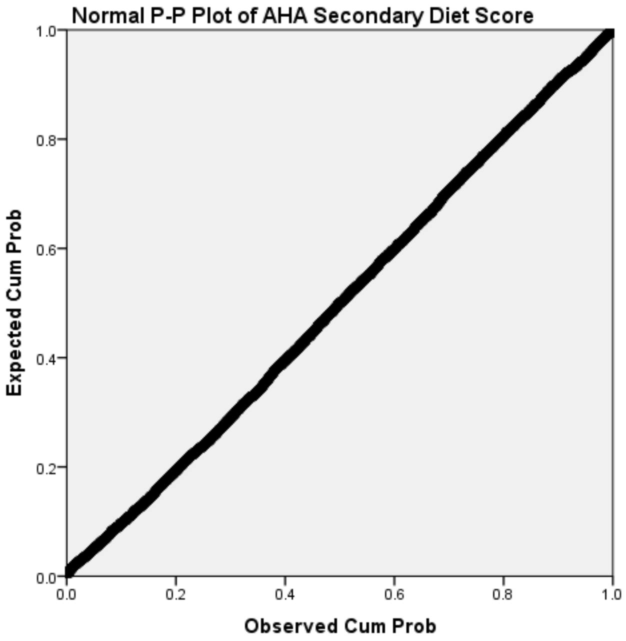

Supplementary Table 1. Descriptive statistics of individual social support variables.

Individual social support variable	Total	Number (%)
Emotional support	n=6776	6183(91.2)
Financial support		4964(73.3)
Attending religious services frequency≥4 times		3972(58.6)
Number of close friends≥4		4262(62.9)
Married or living as married		4179(61.7)

Supplementary Table 2. Cox univariate proportional hazard ratios (HRs) for all-cause and cardio-cerebrovascular mortality by social support score in different models.

Variable	All-cause Mortality			Cardio-cerebrovascular Mortality		
	P	HR	95%CI	P	HR	95%CI
Social support level (As categorical variable)	**<0.001**	0.72	0.67-0.79	**<0.001**	0.65	0.54-0.78
Age (As categorical variable)	**<0.001**	2.21	2.11-2.32	**<0.001**	2.29	2.06-2.54
Male (As categorical variable)	**<0.001**	0.78	0.69-0.86	**0.001**	0.67	0.53-0.86
BMI (As numerical continuous variable)	**<0.001**	0.97	0.96-0.98	**0.004**	0.97	0.95-0.99
Education level (As categorical variable)	**<0.001**	0.77	0.73-0.81	**<0.001**	0.76	0.68-0.86
Race (As categorical variable)	**0.003**	1.11	1.04-1.19	0.21	1.1	0.95-1.29
Cardiovascular disease history (As categorical variable)	**<0.001**	3.35	2.97-3.78	**<0.001**	5.47	4.26-7.03
Cerebrovascular disease history (As categorical variable)	**<0.001**	3.45	2.97-4.00	**<0.001**	4.52	3.32-6.15

CI = confidence interval.

HR = hazard ratios.

BMI = Body mass index.

Bold characters mean a statistical significance.
